# A historical review of experimental imaging of the beating heart coronary microcirculation in vivo

**DOI:** 10.1111/joa.13611

**Published:** 2021-12-14

**Authors:** Neena Kalia

**Affiliations:** ^1^ Microcirculation Research Group Institute of Cardiovascular Sciences College of Medical and Dental Sciences University of Birmingham Birmingham UK

**Keywords:** coronary microcirculation, heart stabilisation, intravital microscopy, ischaemia–reperfusion injury, myocardial infarction, thromboinflammation

## Abstract

Following a myocardial infarction (MI), the prognosis of patients is highly dependent upon the re‐establishment of perfusion not only in the occluded coronary artery, but also within the coronary microcirculation. However, our fundamental understanding of the pathophysiology of the tiniest blood vessels of the heart is limited primarily because no current clinical imaging tools can directly visualise them. Moreover, in vivo experimental studies of the beating heart using intravital imaging have also been hampered due to obvious difficulties related to significant inherent contractile motion, movement of the heart brought about by nearby lungs and its location in an anatomically challenging position for microscopy. However, recent advances in microscopy techniques, and the development of fluorescent reporter mice and fluorescently conjugated antibodies allowing visualisation of vascular structures, thromboinflammatory cells and blood flow, have allowed us to overcome some of these challenges and increase our basic understanding of cardiac microvascular pathophysiology. In this review, the elegant attempts of the pioneers in intravital imaging of the beating heart will be discussed, which focussed on providing new insights into the anatomy and physiology of the healthy heart microvessels. The reviews end with the more recent studies that focussed on disease pathology and increasing our understanding of myocardial thromboinflammatory cell recruitment and flow disturbances, particularly in the setting of diseases such as MI.

## Graphical abstract

Intravital imaging of the beating heart microcirculation in vivo has been challenging. The past 50 years has seen changes in ways to both stabilise and image the heart with a shift from learning about coronary vascular anatomy and physiology to the role of thromboinflammation and other microcirculatory disturbances in cardiovascular disease. This review will discuss the major surgical, technical and imaging innovations that have allowed this to happen.

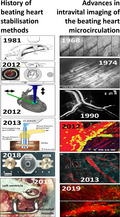

## 1 MYOCARDIAL INFARCTION—REVOLUTIONARY TREATMENT BUT STILL THE LEADING CAUSE OF MORTALITY WORLDWIDE

A myocardial infarction (MI), commonly called a heart attack, occurs when one or more of the major coronary arteries become blocked by a combination of atheroma and blood clots. The resulting ischaemia leads to tissue death and development of a necrotic lesion called an infarct. Treatment focuses on rapidly re‐establishing perfusion which can be successfully achieved with a primary percutaneous coronary intervention (PCI), such as stenting, or by fibrinolysis using intravenous clot‐busting drugs. Revascularisation procedures, particularly primary PCI, have revolutionised the treatment of patients with an acute MI and significantly decreased mortality. However, despite this success, development of heart failure after an MI is the major driver of morbidity and mortality worldwide, placing a huge financial burden on healthcare resources (Cowie, 2017).

Reperfusion, the only way to rescue the ischaemic heart, paradoxically leads to additional tissue damage, a condition termed ischaemia–reperfusion (IR) injury. If IR injury is significant, it can lead to heart failure and even death. This appears to be linked to tissue damage occurring subsequent to inadequate perfusion within the smaller blood vessels of the heart, namely the coronary microcirculation (Bolognese et al., 2004; McAlindon et al., 2018). Indeed, successful restoration of normal blood flow within the occluded epicardial arteries, but with sub‐optimal myocardial perfusion, can be observed in as many as 50% of patients post‐PCI, leading to worse outcomes than in patients with full perfusion recovery (Bolognese et al., 2004; Camici & Crea, 2007; De Maria et al., 2015). This is often referred to as the ‘no re‐flow’ phenomenon and was first described in the brain (Rezkalla et al., 2002). In the heart, no‐reflow occurs when blood is unable to flow in ischaemically damaged myocardial capillaries even when offending occlusions in the coronary artery have been removed. This subsequently enhances the muscle damage caused by the ischaemia alone.

Extensive cardiomyocyte injury can also occur even if flow is resumed within the coronary microvessels. Thromboinflammatory adhesive events, red blood cell congestion, oedema, vasospasm, haemorrhage, endothelial dysfunction and microvascular oxidative damage are amongst the multitude of coronary microcirculatory perturbations that can take place in the initial hours of post‐reperfusion. These events ultimately contribute to extensive tissue damage by reducing functional capillary density which impacts coronary flow and thus deprives cardiomyocytes of essential oxygen and nutrients. Indeed, up to 50% of the final infarct size is considered to be due to reperfusion injury (Yellon et al., 2007). Overall, microvascular impairment post‐reperfusion is an important determinant of subsequent prognosis and is associated with poorer clinical outcomes including larger infarct size, arrhythmias, development of heart failure and higher mortality rates. Current clinical approaches to fully combat myocardial IR injury have been largely unsuccessful (APEX MI Investigator, 2007; Granger et al., 2003) indicating a need for new strategies to limit myocardial damage when blood flow is restored. Indeed, myocardial IR injury post‐PCI remains one of the top 10 unmet clinical needs in cardiology (Fuster, 2014).

## 2 CLINICAL IMAGING OF THE CORONARY MICROCIRCULATION

Increased clinical recognition of the importance of the coronary microcirculation has resulted in the need to identify strategies to improve perturbations within it, and these have gained attention recently (Crea et al., 2014; Luscher, 2017; Pries & Reglin, 2016; Sambuceti et al., 2000). However, clinical research into the role of the coronary microcirculation in cardiovascular disease has been limited. This has meant no efficient strategy to improve microvascular flow post‐PCI has yet been identified. This is due to several challenges which have made it difficult to investigate dynamic events within the coronary microcirculation clinically. The most significant is the fact that the microvessels of interest are small, less than 200µm in diameter, making imaging modalities such as cardiac magnetic resonance (CMR) and positron emission tomography (PET) unsuitable due to their limited spatial resolution (Crea et al., 2014; Pries et al., 2008). This inability to directly image coronary microvessels has led to cardiologists focusing their efforts on improving flow post‐MI within the angiographically visible part of the coronary circulation. Hence, little is known about the full range of coronary microcirculatory responses, particularly at a cellular level, to IR in the clinical setting.

Nevertheless, the potential role of the coronary microcirculation in pathologies of the heart has been implicated since the 1960s. This was around the time that coronary angiography became more widely practiced and it soon became apparent that not all patients with coronary artery disease had obstructions within their epicardial coronary arteries. In 1967, Likoff and colleagues were the first to describe the angiographic absence of coronary artery occlusions in 15 women with angina and abnormal resting and exercising electrocardiograms (ECGs) (Likoff et al., 1967). When the results were presented at the American Heart Association meeting (November 1969), the findings were deemed controversial with some claiming that they were due to the patients suffering from a psychosomatic illness. This lack of coronary artery involvement was thereafter described by more groups and became known as coronary syndrome X, and in later years, microvascular angina, INOCA (ischaemia with no obstructive coronary arteries) and MINOCA (MI with no obstructive coronary arteries) depending on whether symptoms were those of angina or an MI (Arbogast & Bourassa, 1973; Kemp, 1991; Likoff et al., 1967).

In more recent years, progressive loss of distal microvessel perfusion has been clinically demonstrated in patients post‐MI using PET scanning (Thackeray et al., 2015). Furthermore, CMR imaging has demonstrated a dark hypointense core within the infarct zone representing microvascular obstruction in patients with MI which, when persistent, was indicative of patients more likely to develop adverse ventricular remodelling (Bulluck et al., 2018). Functional readouts, such as local blood flow and pressure, determined clinically using a Doppler flow wire inserted into the coronary artery, have also indicated a lack of perfusion in microvessels downstream of an opened epicardial artery. The index of microcirculatory resistance (IMR), a pressure‐temperature sensor guidewire‐based measurement, has also been proposed for quantitatively assessing coronary microvessel functional status indirectly post‐PCI in the territory surrounding the artery from which the measurement is made (Fearon & Kobayashi, 2017). However, these various anatomical and functional assessments are mainly of the larger coronary vessels since IMR measurements cannot be collected from beyond the epicardial artery. Hence, actual visualisation of the coronary microcirculation, let alone any perturbations within them or the vasculoprotective effects of novel therapies, remain impossible to ascertain with current clinical imaging tools.

## 3 HISTOLOGICAL OBSERVATIONS OF CORONARY MICROVESSELS—NOT A DYNAMIC PICTURE

Most of our knowledge of what may be happening within the coronary microvessels post‐IR injury has been obtained from experimental studies in which heart tissue has been interrogated histologically and biochemically for morphological deterioration, thromboinflammatory cell infiltration and infarct size. Various microvascular perturbations noted in tissue sections include the presence of (i) swollen endothelial cells surrounded by cardiomyocytes that are themselves swollen (ii) presence of endothelial gaps (iii) red blood cell (RBC) congestion (iv) platelet and fibrin microthrombi and (v) a high number of intraluminal leucocytes or platelet‐leucocyte aggregates (Ibanez et al., 2015; Sezer et al., 2018; Ward & McCarthy, 1995). However, these one‐time static snapshots cannot indicate which of these events actually reduce or prevent myocardial flow post‐reperfusion, nor can they provide real‐time data on the trafficking kinetics of thromboinflammatory cells in the presence of pathophysiological flow. Therefore, it is not possible to know whether thromboinflammatory cells noted in coronary microvessels in histological sections are actually adherent, occlusive and inhibiting the passage of blood or simply circulating cells or remote emboli that were freely passing through the heart at the time of tissue retrieval. Leucocyte recruitment follows a well‐characterised adhesion cascade which includes crawling, rolling, adhesion, spreading and transendothelial migration. However, histological analysis cannot really ascertain which of these dynamic events are critical in mediating their recruitment in the heart in vivo. Moreover, important microvascular functional information such as the ability of IR injury to modify flow, vessel diameters, functional capillary density, microvessel integrity and leakage are impossible to determine from static sections.

However, experimental imaging of the coronary microcirculation in vivo has remained challenging with the heart's microcirculation often being referred to as a research ‘black box’ (Pries & Reglin, 2016). A number of factors have confounded in vivo microscopic imaging of the heart which include its deep anatomical location in the chest wall, low transparency, and the presence of motion inherent to the beating organ and from nearby lungs. Indeed, of all the major organs, the heart has the highest maximum motion velocity under normal physiological conditions (up to 19.9 mm/s), which can increase almost 2.5 fold when mice are placed on ventilation (47.8 mm/s) (Lee et al., 2017). As a result of this movement, the heart has remained elusive to in vivo imaging techniques such as fluorescent intravital microscopy (IVM). IVM is a powerful tool used for real‐time imaging of the microcirculation with cellular resolution in anaesthetised animals and, therefore, under conditions closely approximating those of a natural environment. It is largely based on the detection of fluorescence used to identify various cellular structures such as circulating blood cells, endothelial cells and even proteins such as albumin and coagulation factors. Indeed, rapid technological improvements permitting near‐simultaneous imaging of multiple fluorescent signals in the same animal, and advances in fluorescence tagging methods, have enabled more specific cell types and their spatial‐temporal interactions to be determined. Over the decades, this technique has been applied to a number of healthy vascular beds and in experimental models of disease and injury, notably the cremaster muscle (Nolan et al., 2008), kidney (White et al., 2013), liver (Kavanagh et al., 2010), stomach (Kalia et al., 2002) and small intestine (Kavanagh et al., 2013) and colon (Yemm et al., 2015). However, the development of unique methodological approaches for visualising and manipulating the coronary microcirculation lagged behind imaging of the microcirculation in other solid organs. Indeed, data generated in transparent tissues such as the cremaster, a skeletal muscle surrounding the testicle, have often been extrapolated to the heart. However, it is clear that such extrapolations should be avoided in light of increasing evidence of site‐specific microcirculatory perturbations, and molecular mechanisms governing these responses, even to similar injuries.

## 4 THE EARLY YEARS—A FOCUS ON ANATOMY AND PHYSIOLOGY (1968–1980s)

**FIGURE 1:**
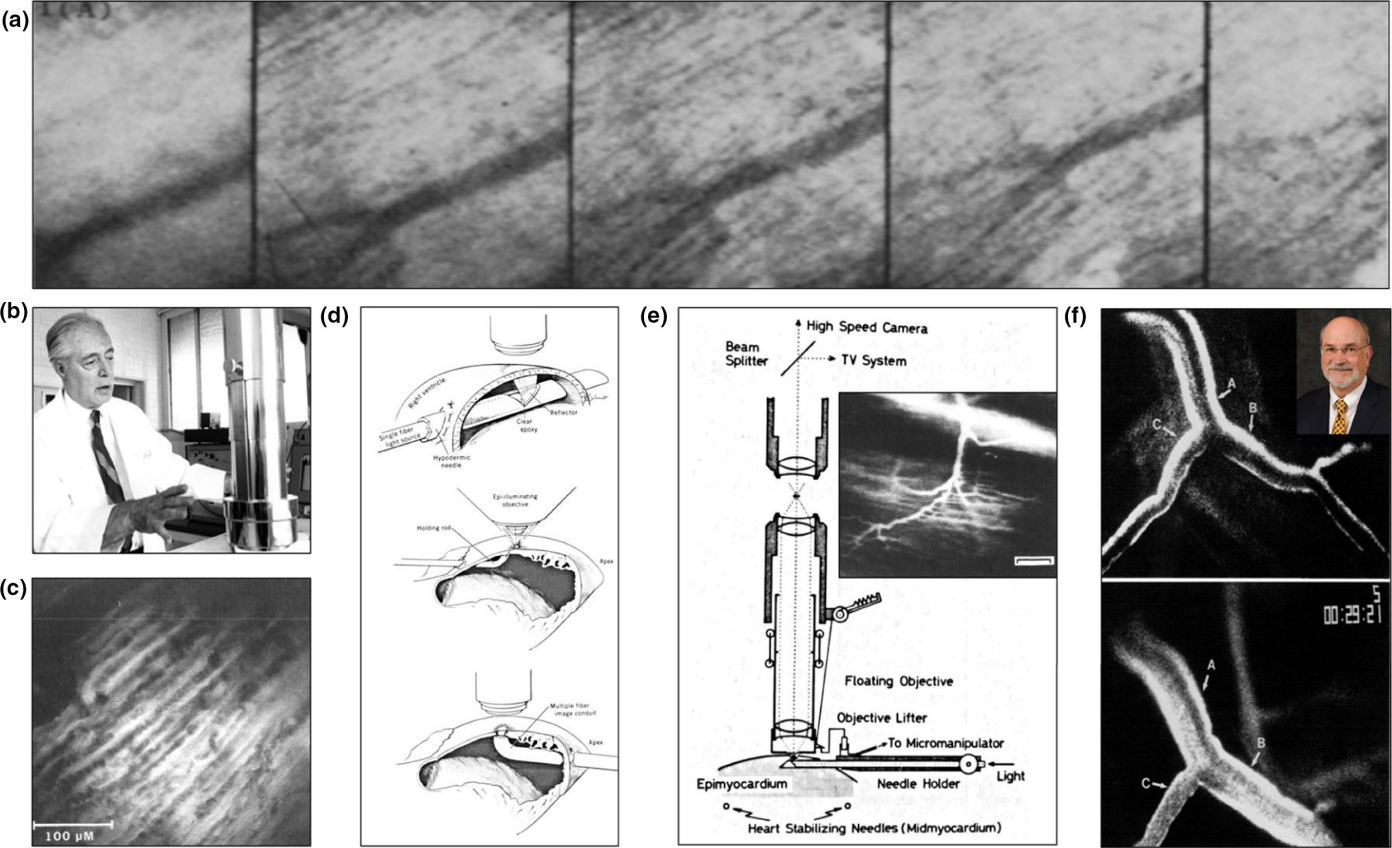
Early attempts at intravitally imaging the beating heart coronary microcirculation in vivo*—*1968–1980s. (a) The first published intravital image of the rat beating heart in a study by Martini and Honig in 1968. A series of sequential frames are shown to illustrate how the movement of the heart during a cardiac cycle affected image focus, with authors pointing out that only the third image was suitable for subsequent analysis. These early captures were not of a good enough resolution to determine the direction of blood flow. Reproduced from Martini & Honig, 1969. (b) Eminent cardiologist, Professor Richard J. Bing, pictured in 1970. His seminal work was the first to image and quantitate the phasic blood flow in the heart microcirculation in response to the systolic and diastolic phases of the cardiac cycle. (c) An intravital study led by Professor Bing showing the anatomical arrangement of the capillaries in the turtle ventricle to be primarily parallel and concurrent, but with some intercapillary anastomoses present. Reproduced from Tillmanns et al., 1974. (d) Images highlighting the various ways in which the rabbit heart could be stabilised with a holding rod or kept ‘motion free’, and trans‐illuminated either from beneath the imaged microvessels with a light source inserted into the muscle, or from above using conventional epi‐illumination. Reproduced from Nellis et al., 1981. (e) Intravital studies led by Professor Tamotsu Takishima used a ‘floating objective’ combined with intramuscular trans‐illumination of the canine heart which was held in place using stabilising needles. The inset shows an image of an epimyocardial terminal arteriole and its branching capillaries, obtained by fluorescent intravital microscopy. Scale bar = 100 μm. Reproduced from Ashikawa et al., 1986. (f) The 1980s was dominated by beating heart intravital research led by Dr William Chilian and colleagues. These images of canine coronary microvessels were obtained by fluorescence microscopy after FITC‐dextran infusion either under baseline conditions (upper panel) or during adenosine infusion (lower panel) to demonstrate vasodilation of various diameter microvessels. In the top panel, a parent vessel (a) and two daughter branches (b and c) with diameters of 153, 130 and 104 μm, respectively, are shown. The lower panel shows vessels a, b and c vasodilating to 170, 162 and 111 μm respectively. Reproduced from Chilian & Layne, 1990

The earliest intravital studies of the heart focussed primarily on increasing our understanding of the anatomy of the coronary microvasculature and its physiological regulation of blood flow. Seminal studies investigated the effects of the systolic/diastolic phases of the cardiac cycle, hypoxic conditions and vasoactive mediators on microvessel perfusion and tone. The first published intravital study was led by Jeannine Martini and Carl Honig from New York in 1968 in which they imaged the most superficial layers of the exposed beating heart of ventilated rats using, by today's standards, rather crude and laborious methods (Martini & Honig, 1969). A flat portion of the right ventricle of the freely beating heart was imaged after epi‐illumination with a point‐source strobe light, collected using an objective with a long enough working distance to prevent its contact with the beating heart. A glass coverslip, placed over the ventricle to prevent glare, did little to control the excessive movement and so images were only taken at the end of expiration when respiratory movement briefly stopped. Whilst this early attempt at in vivo imaging of the beating heart is applaudable, the technology at the time meant that 100–150 feet of film was needed to capture only 30–50 focussed frames, many of which were not useable (Figure 1a). A laborious analysis method involved focussed frames being projected in a darkened room onto a sheet of white paper using a projecting microscope calibrated with a micron grid. Although captured images were just about sufficient to differentiate capillaries from muscle, they were not good enough to determine the direction of blood flow. Nevertheless, the study provided novel data including the interesting observation that only about 50% of available coronary capillaries in the unstressed heart were visibly perfused, but more rapidly opened when hypoxia was induced by changing the percentage of oxygen inspired.

In the next decade, eminent cardiologist Professor Richard Bing (Figure 1b) and colleagues from California were the first to directly image in vivo the phenomenon of a phasic blood flow velocity pattern in arterioles and capillaries bought about by systolic and diastolic phases of the cardiac cycle. Their intravital study used high‐speed cameras to capture images from the left atrium of cats (Hellberg et al., 1972). The atria were used as they presented less motion than the ventricles, although not soon after, their system was adapted in order to image the coronary microcirculation of canine and turtle (because of a lower heart rate) ventricles (Tillmanns et al., 1974). For both atrial and ventricular imaging of turtle hearts, a new method of trans‐illumination was developed, which included shining a light through the heart muscle, rather than on the heart by epi‐illumination. This involved a rather invasive procedure in which the muscle was pierced, just below the outermost epicardial layer, with a light‐carrying 20‐gauge microneedle. Light transmitted through the atrial or ventricular wall illuminated the overlying muscle and microvasculature. They also developed a novel method to keep acquired images in focus despite changes in the vertical distance between the moving turtle heart surface and the stationary microscope objective. This consisted of a ‘floating’ objective which moved in unison with the beating heart. Microcinematography (today called time‐lapse photography) was used to take photographs at 440 frames/second. This rate of filming made it possible to track a RBC in transit frame‐by‐frame. Novel insights provided a demonstration that in the canine heart, the inside diameters of arterioles and venules ranged from 20–36 and 15–29 μm, respectively, during diastole but decreased to 15–29 and 11–20 μm, respectively, during systole. Also, maximal flow was observed in arterioles during diastole but was decreased in systole. However, the opposite was noted in capillaries and venules whereby RBC velocity was maximal during systole. The explanation provided was that ventricular contraction ‘throttled’ blood flow in arterioles thereby decreasing RBC velocity, but at the same time ‘massaged’ blood flow in the compressed capillaries towards the venules, thereby increasing capillary RBC velocity. Additional anatomical findings demonstrated that capillaries lay on either side of muscle fibres, with approximately one capillary per fibre, and were primarily parallel in arrangement but with several interconnections between them that could be observed at higher magnifications (Figure 1c). Since it was not possible to keep the microcirculation in focus during all phases of the cardiac cycle, clear visualisation of finer structural arrangements of the capillary bed was not possible.

Further information on the haemodynamics of the coronary microcirculation, again focussing on the effect of myocardial contraction on blood perfusion, was provided in 1981 by Nellis and colleagues from Pennsylvania (Nellis et al., 1981). They intravitally imaged the rabbit heart and compared three different protocols in which the heart was either fixed in place by a holding rod that was passed through the ventricular chamber and trans‐illuminated from within, fixed in place and epi‐illuminated from the above objective, or trans‐illuminated from within but in a ‘motion‐free’ non‐fixed heart (Figure 1d). Preferring to limit external restriction or stabilisation of the heart as little as possible, they successfully imaged the ‘motion‐free’ heart by ingeniously linking ECG‐gating to a computer‐controlled stroboscopic light source (produces flashes of light). Gating is one of the oldest and most straightforward ways of effectively reducing cardiorespiratory artefacts by synchronising image acquisition with certain periods of the cardiac and/or respiratory cycle. In this study, a short‐lived illumination was triggered at the same point in the cardiac cycle, namely during the QRS complex, which gave the perception of tissue stillness. The major finding was of a phasic relationship between vessel diameter and ventricular pressure, but this was only tested in two epicardial coronary veins. Possibly as a result of these low numbers, no consistent trend was observed with both increases and decreases in vein diameter noted during systole. Arterioles were not examined as they were of a short length due to the fact that they became subepicardial as they penetrated the muscle wall. Limited quantitative data were provided, which was focussed on large blood vessels of the heart. Moreover, no intravital images were presented and so it is difficult to ascertain the real success of this method at resolving these vessels or the smallest coronary capillaries.

Compensating for cardiac and respiratory‐induced motion with minimal trauma to the heart remained a challenge for intravital studies throughout the 1980s. In this decade, the field was dominated by the work of American cardiovascular physiologist, Professor William Chilian (Chilian et al., 1986, 1987, 1989). A computer‐controlled stroboscopic light source was again used, which briefly illuminated the cat or dog heart at the same time point during late diastole, to give the impression of the epicardial microvasculature being motionless. This was later combined with high frequency ventilation synchronised to the cardiac cycle. Since tidal volume was small, there was little respiratory effect on cardiac motion. These studies also partially restrained the canine heart using four 22‐gauge needles attached to a rod inserted into the heart. The myocardium within this stabilised region was still able to contract vigorously but vertical motion was somewhat limited without compromising blood flow or traumatising the microvasculature (Chilian & Layne, 1990). The plethora of work published by this group provided further novel anatomical and physiological insights, particularly on the role of the coronary microcirculation in autoregulatory control and functional hyperaemia. These included demonstrating that autoregulatory responses occurred primarily in arterioles less than 150 μm in diameter, which vasodilated during reductions in coronary perfusion.

Continuing the theme of imaging the stabilised heart of large mammals, the same decade also saw a team led by Professor Tamotsu Takishima in Japan image the coronary microcirculation of the canine ventricle (Ashikawa et al., 1984, 1986; Komuru et al., 1990). Excessive horizontal movement was again limited using steel needles inserted into the ventricle mid‐myocardium and held in place by a holder. Epimyocardial or surface microcirculation was visualised by trans‐illumination using a system not too dissimilar to that described by Bing and colleagues in the previous decade, involving a light‐conducting glass fibre inserted into the sub‐epicardial muscle layer to illuminate the overlying tissue. They developed a different ‘floating’ objective, which included an additional convex lens placed between the heart and the microscope objective, capable of moving in unison with cardiac motion (Figure 1e). The most significant accomplishment of this study was the fact that it acquired images continuously throughout the cardiac cycle, rather than at particular time points. These were of sufficient quality to detect small changes in RBC velocity and microvessel diameters. The latter was possible as studies led by Takishima, and also by Chilian, combined the use of a fluorescent microscope and systemic injection of fluorescein isothiocyanate (FITC)‐labelled dextran, to measure changes in arteriole, capillary, and venule diameters in response to vasoactive mediators (Ashikawa et al., 1986; Chilian & Layne, 1990; Figure 1e, f). Indeed, this was used to show for the first time that neuropeptide Y could vasoconstrict canine coronary arterioles in a dose‐dependent manner and thus play an important role in pathological conditions such as myocardial ischaemia (Komaru et al., 1990).

Collectively, these early studies provided unique insights into the anatomy of the coronary microcirculation, allowing visualisation and measurement of vessels as small as 10–15µm in the natural beating heart environment. Physiologically relevant changes in vessel diameters, RBC velocity and the flux of fluorescently labelled macromolecules could be assessed, but also in response to various pharmacological agents and vasoactive stimuli. These pioneering researchers, who did not have access to the current modern imaging tools, were not deterred by the technical challenges that faced them. They published new concepts that enhanced our knowledge about basic control systems regulating myocardial perfusion at the level of the microcirculation. However, the following two decades saw a significant decline in intravital imaging studies of the intact beating heart microvasculature. It is not clear why this was the case but may have been linked to the fact that the use of large mammals in research was becoming increasingly unpopular and in vitro models, such as the ex vivo Langendorff perfused heart, endothelial cell culture assays and flow cytometry, gained popularity. These were cheaper to run, technically easier to perform than the complicated surgery required for the beating heart research and, importantly (for the researcher), generated more data than in vivo research in a shorter space of time.

## 5 THE LATER YEARS—A FOCUS ON CARDIAC DISEASE PATHOLOGY (2012–2021)

It was not really until a decade into the new century that there was a noticeable renewed interest in intravital imaging of the beating heart microcirculation. The modern era saw newer methods to stabilise the heart including modifications to compression with a coverslip (Li et al., 2012), gluing a stabiliser on to the heart surface **(**Kavanagh et al., 2019a; Lee et al., 2012) or using suction‐based devices (Jung et al., 2013; Vinegoni et al., 2012). However, it was the introduction of more advanced microscopy, such as laser raster scanning confocal, spinning disk confocal and multiphoton imaging, which really progressed the field, along with the use of new fluorescent probes and reporter mice. Unlike the earlier work, these new studies focussed less on anatomy and physiology of the healthy coronary microcirculation but aimed to either technically improve spatiotemporal resolution or to investigate the coronary microcirculation in the context of diseases such as MI / IR injury. Thromboinflammatory cell involvement was known at this time to be critical in IR injury in many other organs, including the liver (Oliveira et al., 2018), kidneys (White et al., 2013), gut (Kavanagh et al., 2015) and brain (Vital et al., 2016). Hence, there was a specific interest in developing protocols to intravitally image the dynamic behaviours of these cells in the heart.

**FIGURE 2:**
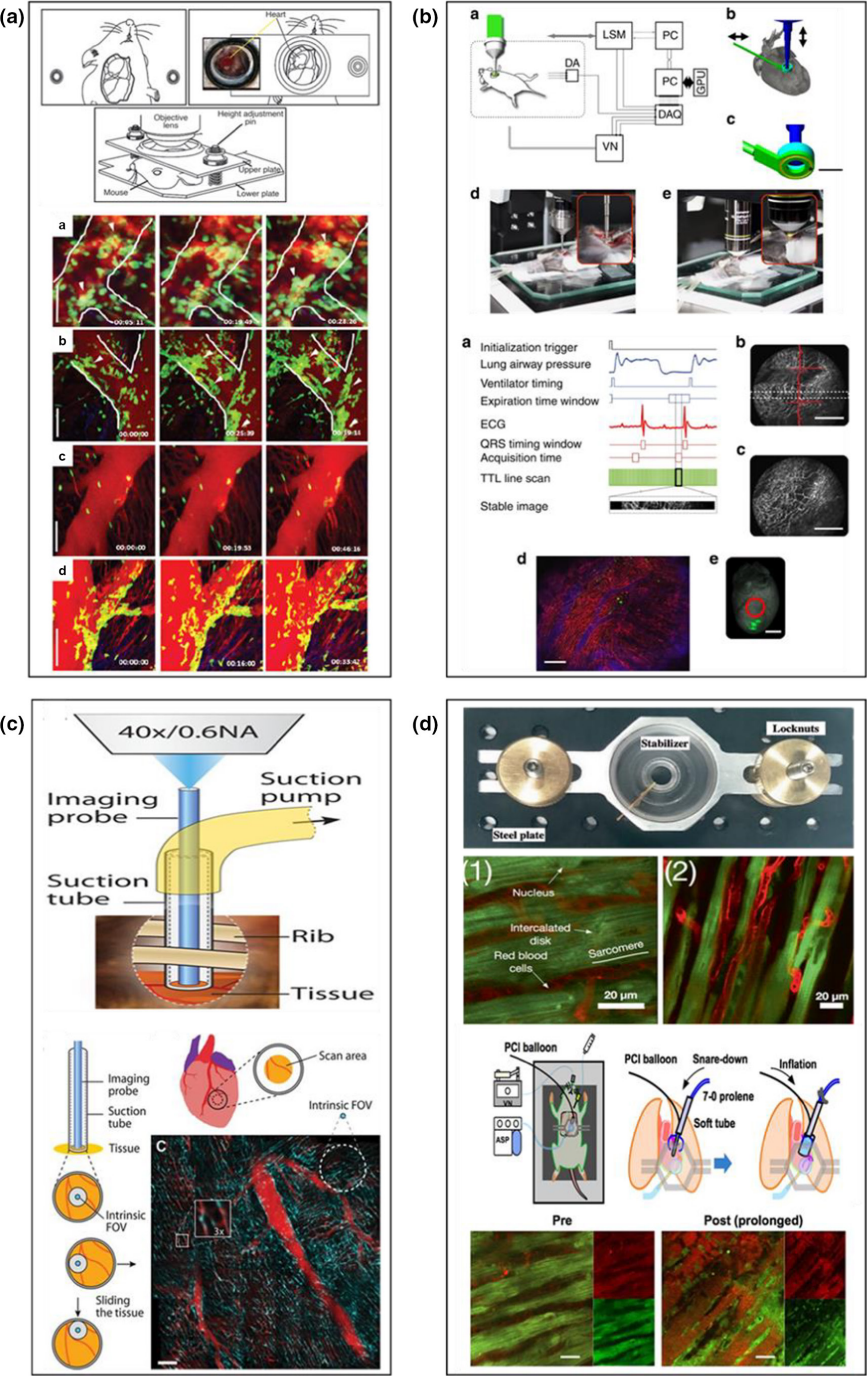
Recent attempts at intravitally imaging the beating heart coronary microcirculation in vivo*—*2012–2018. (a) Imaging a beating donor heart transplanted into the neck region of a recipient mouse. A ring of Vetbond was applied to the bottom of the cover glass portion of the stabilisation plate and held against a region of the left ventricle. The lower panel shows multiphoton microscopy images of the donor heart transplanted into LysM‐GFP mice in which infiltrating host neutrophils appear green. Reduced neutrophil recruitment is noted in ICAM‐1 deficient mice (third set of images). Blood vessels are labelled with Q‐dots injected intravenously before imaging. Scale bars = 60 μm. Reproduced from Li et al., 2012. (b) An alternative glue method, which avoided compression from an overlying cover glass, involved the underside of a ring stabiliser being bonded to the surface of the mouse beating heart. A shallow groove (yellow ring) was carved out to increase contact area between the stabiliser and the bonding material, and thus ensure better attachment to the heart. The stabiliser was filled with saline, needed for the water‐immersion objective and also to prevent the underlying myocardium from drying out. An extremely thin (1.3 mm diameter) ‘stick’ microprobe x6 objective was used to gain access to the centre of stabiliser and capture large field of view images. The lower panel shows how gated images were reconstructed from those taken during specific points in the cardiac and respiratory cycles. A raw image (b‐b) shows the extent of the motion artefact which is significantly improved in the reconstructed imaged (b‐d). DA, differential amplifier; DAQ, data acquisition card; ECG, electrocardiogram; GPU, graphic processing unit; LSM, laser scanning microscope; VN, ventilator. Scale bar = 100 μm (b‐b, b‐c); 200 μm (b‐d) and 2 mm (b‐e). Reproduced from Lee et al., 2012. (c) A minimally invasive method for imaging the mouse beating heart used an in‐house designed miniature steel microendoscope lens probe—20 mm length; 1.25 mm diameter—advanced towards the heart through a small incision in the skin and fourth left intercostal space. The bottom two‐thirds of the probe is housed within a steel sleeve, surrounded by an outer steel tube attached to a suction pump. The probe transmitted a signal to an overlying objective lens. The lower panel shows how ‘gliding’ the suction tube on the heart surface allowed a larger scan area to be obtained. A mosaic image of the blood vasculature of the left ventricle identified using GFP protein in Tie2^+^ cells (cyan) and an injected anti‐CD31 antibody conjugated with Alexa 647 (red). Scale bar = 100 μm. Reproduced from Jung et al., 2013. (d) Heart stabiliser and plate designed for use in rats. The sunken central hole, measuring 8 mm, was covered with a round cover glass and attached to the heart using suction (thin yellow suction tube visible in the stabiliser centre). The entire steel contraption was fixed in place using tightening locknuts on the bilateral arms. Two‐photon microscopy was used to visualise GFP^+^ cardiomyocytes (green) and blood flow through the capillaries. Blood flow is stained with red‐labelled dextran and the endothelium with red‐labelled isolectin B4. The lower panels show how a balloon catheter, used clinically for primary PCI, was placed at the base of the left atrial appendage and held in place using a suture. Remote manual inflation‐induced occlusion of the LAD coronary artery, and therefore left ventricular ischaemia, with deflation inducing reperfusion injury. This allowed the heart to be imaged during both ischaemia and immediately upon reperfusion. The visible leakage of dextran‐red from the vessels onto the myocytes provides a reliable indicator of microvascular leakage (different periods of ischaemia were examined in this study (15, 30 and 60 min)—the term ‘prolonged’ in the image refers to the longer 60‐min period of ischaemia). Scale bar = 20μm. Reproduced from Matsuura et al., 2018a/b

Cardiothoracic surgeon, Professor Daniel Kreisel from Missouri started off this new and exciting era in 2012 with a multiphoton‐based intravital study to image leucocyte trafficking in the mouse beating heart (Li et al., 2012). Unlike previous studies, their research involved imaging transplant‐mediated IR injury in donor hearts, reperfused with host blood, after being transplanted into the neck region of syngeneic mice. The recipient was either a LysM‐GFP^+^ reporter mouse, in which endogenous neutrophils expressed high levels of GFP and were therefore brightly labelled (NB. monocytes/macrophages labelled to a lesser extent and therefore dimmer), or a CX_3_CR1‐GFP^+^ reporter mouse whose monocytes expressed high levels of GFP. Elegant surgery connected the donor ascending aorta and pulmonary artery to the recipient's right common carotid artery and jugular vein, respectively, after which the donor heart resumed a regular heartbeat immediately following reperfusion. A small ring of Vetbond, a veterinary adhesive, was applied to the bottom of a coverglass contained within a stabilisation plate, and briefly held against the left ventricle of a partially exteriorised donor heart to secure it (Figure 2a). To label the endothelial cells, fluorescent quantum dots (Q‐dots) were used. Unlike commonly used organic fluorescent dyes, Q‐dots are inorganic nanocrystals of extreme brightness and highly resistant to photobleaching. GFP‐labelled neutrophils / monocytes and Q‐dots were excited using a multiphoton laser beam tuned to 890 nm and time‐lapse images captured. To reduce tissue motion, Z‐stack acquisition was synchronised with the heartbeat to yield stable videos of superficial structures such as coronary veins. Imaging was initiated 1 h after transplantation. Although the images and videos provided are of excellent quality, it is surprising that hardly any contractile motion was evident. This may be linked to the fact that stabilisation involved compressing the heart with the coverslip and essentially ‘squeezing’ the heart to reduce motion. Having said that, the authors claimed this ‘exerted virtually no direct pressure on the heart’.

Interestingly, minimal neutrophil recruitment was observed in post‐capillary venules, which is commonplace in other IR injured and inflamed organs. This event was noted primarily in larger superficial coronary veins, where neutrophil crawling and clustering were noted. The kinetics of monocyte recruitment was similar, although intravascular clustering was not identified. To investigate the adhesive mechanisms involved, studies were conducted in hearts from donor mice with mutant intercellular adhesion molecule‐1 (ICAM‐1) or in host mice pre‐treated with antibodies to leucocyte integrins that interact with endothelial ICAM‐1, namely LFA‐1 (α_L_β_2_ or CD11a/CD18) and MAC‐1 (α_M_β_2_ or CD11b/CD18). Reduced neutrophil crawling velocities, clustering and extravasation was noted in the absence of ICAM‐1 and Mac‐1, with LFA‐1 blockade preventing neutrophil adhesion and extravasation. These adhesion molecules have previously been demonstrated histologically and flow cytometrically to be important in myocardial leucocyte infiltration (Meisel et al., 1998; Palazzo et al., 1998). However, this novel in vivo study was the first to directly image their role in the beating heart and describe changes in dynamic behaviours, such as rolling and crawling, as well as define the anatomical component of the vasculature in which this happened. Spatial resolution appeared sufficient with multiphoton microscopy to allow transmigration from venules to also be easily identified. Since the visualised GFP^+^ fluorescent cells were those that came from the host, this heterotopic heart model offered the advantage of imaging the kinetics of only infiltrating cells and not resident unlabelled donor cells.

This compression method for stabilising the heart was adapted to also image the native heart in its normal intrathoracic position after myocardial IR injury was induced by reversible ligation of the left anterior descending (LAD) coronary artery. No pacing of the heart was performed and mice were ventilated with room air but, nevertheless, were able to tolerate being imaged for at least three hours. Neutrophil recruitment was not noted in the absence of injury. Importantly, native and donor hearts showed similar neutrophil kinetics after reperfusion. It is for this reason that the group later continued with the transplant‐mediated IR injury model to define the upstream signals that orchestrated neutrophil trafficking (Li et al., 2016). They demonstrated an important role for tissue‐resident CCR2^+^ macrophages in promoting the extravasation of neutrophils through TLR9/MyD88‐mediated production of CXCL2 and CXCL5 chemokines. By depleting resident cardiac macrophages in donor mice using clodronate liposomes prior to transplantation, they demonstrated that although neutrophils slowed down and adhered to the vessel, they failed to extravasate into the myocardium efficiently. Further elegant studies were conducted in transplanted donor hearts in which CCR2^+^ macrophages were depleted using diphtheria toxin, or in MyD88‐deficient mice, to show the importance of this specific sub‐population and signalling pathway respectively. This seminal paper is the first to dissect several mechanisms involved in mediating neutrophil to the transplant induced‐IR injured coronary microcirculation in vivo. The advantage of the cardiac transplant model is nicely exemplified by the fact that it allowed depletion of certain cell populations and selective controlling of the expression of molecules in resident cells of the donor heart. Most recently, the group used this model to demonstrate a role for ferroptotic cell death and TLR4/Trif signalling in initiating neutrophil recruitment to the injured beating heart in vivo (Li et al., 2019).

Two publications from the group of radiologist and imaging scientist Professor Ralph Weissleder in Massachusetts were also published in 2012. Confocal and multiphoton microscopy was used but with a much smaller stabiliser than previously described, and combined with image acquisition triggered using retrospective ECG‐gating at appropriate phases of both the respiratory and cardiac cycles (Lee et al., 2012; Vinegoni et al., 2012). In the first of these method papers, the stabiliser comprised a 3‐cm‐long rigid metal arm with a flat stainless steel small ring at one end that had an outer and inner diameter of 3.6 and 2.2 mm respectively (Lee et al., 2012). A micromanipulator was used to carefully position it on the mouse heart, after which it was glued using Dermabond to the epicardium, providing a contact‐free, non‐compressed central area subsequently filled with saline. The only pressure was the one induced by the heart itself while contracting against the stabiliser ring, which the authors described as being similar to the heart beating against the chest wall. An extremely thin (1.3 mm diameter) ‘stick’ microprobe x6 objective was used to gain access to the centre of stabiliser and capture large field of view images. Initially, the authors attempted imaging at certain time points in the cardiac cycle but this still resulted in respiratory motion artefacts. Thereafter, two points on both cardiac (15 ms after P wave on an ECG) and respiratory (90 ms near the end of expiration) cycles were chosen to trigger a more advanced acquisition of images (Figure 2b). An algorithm was designed for these time points and images with minimal motion artefacts were reconstructed. A custom‐built Faraday cage was built to remove electromagnetic noise during ECG recordings. This set‐up was then tested in a model of myocardial IR injury to image rhodamine 6G‐labelled leucocytes, with rolling noted, for the first time, in coronary capillaries. The description of leucocyte trafficking in this study was basic and minimal analysis was performed on the trafficking events described. Interestingly, Hoechst 33258 was applied topically to the heart surface to identify cardiomyocyte nuclei.

The second methods‐based paper from this group offered a different stabilisation approach, modified to not only allow more than one region of the mouse heart surface to be imaged, but also more reproducibly so that it could be easily gated (Vinegoni et al., 2012). A miniaturised suctioning stabiliser was designed consisting of an internal 2 mm diameter chamber and external 4.5 mm diameter chamber. A vacuum was maintained in the external chamber through the use of a 1 mm diameter conduit that connected it to a vacuum regulator. A negative pressure of 50–60 mm Hg was applied to attach it to the heart surface. The stabiliser size meant that only a small surgical incision was required to gain access to the heart, but also meant that only a stick objective could be used to enter the inner chamber in order to acquire images. Since glue was not used to permanently fix the heart to one location, the suction pressure could be released to allow the stabiliser to be moved and attached to different parts of the heart in order to sample different areas. The stabiliser did not compress the heart nor block its motion during contractions and actually permitted it to move with a reproducible motion that could more easily be gated. Image acquisition and reconstructions were again performed using a triggering or retrospective gating approach. Comparisons were made between their earlier coverslip compression method and this newer suction method. The images provided clearly showed distortions with the former. Suction did not compromise tissue integrity and no anatomical damage was present in the microvascular network on the surface of the heart in direct contact with the stabiliser. In the following years, this group modified their method, which included the development of retrospective gating algorithms for segmented microscopy (Vinegoni et al., 2013) and the ability to resolve subcellular structures within cardiomyocytes (Aguirre et al., 2014). They also published an extensive methods paper, reviewed their models, and distributed the 3D STL files (file type used in 3D printing) so that research groups could 3D print the stabilisers (Vinegoni et al., 2015a, 2015b).

Suction was also a key component of an elegant microendoscopic probe‐based method described by a study lead by Professor Seok H Yun, an optics expert in Boston (Jung et al., 2013). This paper was one of the first to use the beating heart imaging model to generate significant data on the nature of immune cell infiltration to the infarcted mouse heart. An in‐house designed miniature steel microendoscope lens probe, with a 20 mm length and 1.25 mm diameter, was advanced towards the heart through a small incision in the skin and fourth left intercostal space, without any ribs being broken. The bottom two‐thirds of the probe was housed within a steel sleeve surrounded by an outer steel tube attached to a suction pump. The probe transmitted a signal to an overlying objective lens (Figure 2c). A suction pressure of 50 mm Hg reduced heart movement to <5–10 μm without affecting the flow of labelled RBCs. Histology demonstrated no signs of tissue damage after prolonged suction for 1 h at this pressure. Interestingly, tissue damage was not even evident when the probe and suction tube was ‘glided’ over the heart surface in order to gain a wider area of image capture. This meant that although the probe only imaged a 250 μm field of view, a mosaic high‐resolution image of 1.2 mm^2^ could be obtained (Figure 2c). A significant advantage of minimally invasive endoscopic imaging was the fact that the small chest incision could be easily suture closed, the mouse left to recover and the heart imaged longitudinally over a period of days.

*Cx_3_cr1^gfp^* ^/+^ and *LysM^gfp^* ^/+^ mice were again used to track CX_3_CR1^+^ monocytes and LysM^+^ neutrophils (monocytes also labelled, but would appear dimmer than brightly labelled neutrophils), respectively, to the infarct, which was induced by permanent LAD coronary artery ligation. By sliding the probe over the heart, myeloid cell infiltration into both the infarcted and remote areas could be analysed. The number of LysM^+^ neutrophils and CX_3_CR1^+^ monocytes remained low in non‐infarcted tissue but both cell types infiltrated the infarcted tissue within hours of ligation. Interestingly, monocyte recruitment was higher, remained elevated over days and more rapid than that of neutrophils. This rapid response was in contrast to the generally accepted notion that neutrophil recruitment preceded that of monocytes in MI. Additional insights into the velocities of circulating and rolling monocytes in large coronary arterioles and venules were also presented. An unexpected abundance of monocytes crawling or slowly rolling on the vessel walls was also noted in the heart, which the authors concluded were monocytes ‘patrolling’ coronary vessels during steady‐state immune surveillance. This was the first direct evidence that active immune surveillance mechanisms existed in the heart. Indeed, it is now accepted that there are at least two mouse monocyte subsets, namely inflammatory (Ly‐6C^hi^CCR2^hi^CX_3_CR1^lo^) and patrolling (Ly‐6C^lo^CCR2^lo^CX_3_CR1^high^) monocytes. Inflammatory monocytes accumulate at sites of inflammation, where they can differentiate into macrophages or dendritic cells. However, patrolling monocytes survey the vasculature by constantly crawling along the endothelial surface and are therefore early responders to sterile inflammation and tissue repair (Kratofil et al., 2017). This phenomenon has also been described for neutrophils in the lungs where ‘crawling’ neutrophils provided the first line of defence against infection in pulmonary capillaries (Harding et al., 2014). Jung and colleagues concluded from their novel beating heart observations that the patrolling subset was the main source of the monocytes found in the infarct immediately after MI, thus explaining their rapid response. It is clear that without real‐time, in vivo imaging of the beating heart coronary microcirculation, these novel discoveries on the dynamics of resident and flow‐captured leucocyte sub‐sets would not have been possible.

Matsuura and colleagues from Osaka, Japan also developed a non‐gated beating heart imaging method (Matsuura et al., 2018a). However, they used two‐photon microscopy and a custom‐built cardiac surface stabiliser to image the rat heart (Figure 2d), which they believed provided greater stability than all previously described methods and thus allowed resolution of even sub‐cellular structures such as the mitochondria within individual cardiomyocytes. In order to attach a much larger stabiliser, the entire anterior chest wall was resected to expose the left ventricle. CAG/GFP transgenic Lewis rats, which ubiquitously expressed the green fluorescence protein gene under the control of the CAG promoter, allowed cardiomyocytes to be observed. This was combined with systemic infusion of Alexa 568‐conjugated to isolectin B4 to detect endothelial cells, and Texas red‐labelled dextran to detect blood flow. This method was subsequently used by the group to image suppression of the contraction/relaxation cycle, increased leucocyte presence and cell permeability in IR‐injured rat and mouse hearts (Matsuura et al., 2018b). Manual tying and untying of the LAD artery alongside simultaneous image acquisition is technically not possible. Therefore, to capture the events taking place during the ischaemic period, and in the immediate aftermath of reperfusion, a clever method was used in which a suture‐held clinical PCI balloon was remotely inflated above the LAD coronary artery to induce ischaemia, and then deflated to induce reperfusion. The videos provided are of an excellent quality for observing the anatomy and patchy loss of GFP fluorescence in sarcomeres and mitochondrial membranes of myocytes during ischaemia, indicative of cell death. GFP‐positive regions showed stronger cyclic contractions than dead regions. The visible leakage of systemically injected dextran‐red from the vessels onto the myocytes also provided a reliable indicator of microvascular leakage (Figure 2d). Despite using in‐house software to eliminate motion, significant motion is still evident in the videos making detection of microcirculatory functional aspects such as GFP^+^ leucocyte trafficking difficult. Most recently, this group used this method to image non‐vascular structures. Indeed, Masuyama and colleagues provided the first intravital evidence of altered cardiomyocyte alignment in mice with dilated cardiomyopathy compared to those with healthy hearts (Masuyama et al., 2021). The cardiomyocytes were visualised in *Rosa26^mT^* ^/^ *^mG^* reporter knock‐in mice (*mT*/*mG* mice) in which the cardiomyocyte membrane was double fluorescently labelled with tdTomato and eGFP and then imaged using multiphoton microscopy.

**FIGURE 3:**
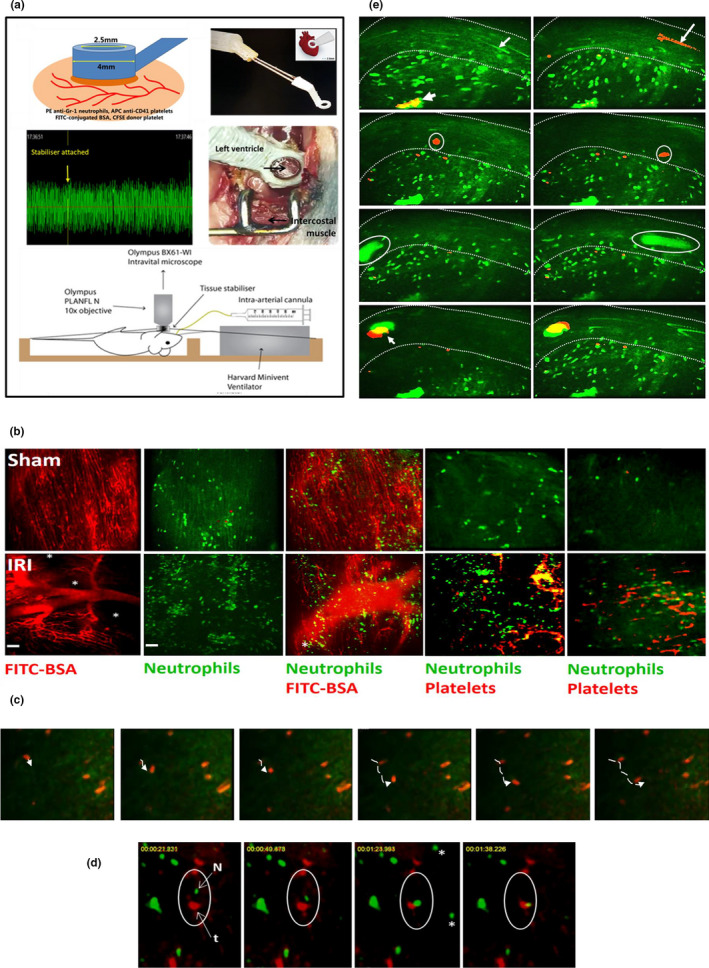
Imaging multiple dynamic coronary microcirculatory perturbations post‐IR injury in the beating mouse heart—a 2019 study. (a) A small in‐house designed 3D printed stabiliser is vet‐bonded to the mouse beating left ventricle and imaged in its centre. The photoplethymosograph shows BP (and indeed HR) remained constant after the stabiliser was attached. An Olympus BX61‐WI microscope, with spinning Nipkow confocal capabilities, is used to capture images with a x10 dry objective and an EMCCD camera. (b) In sham mice, an extensive network of FITC‐BSA‐perfused capillaries can be observed, which parallel the arrangement of muscle fibres, with cross connections that run obliquely to muscle fibres along their length. However, blood flow is compromised in IR‐injured hearts as evidenced by not all capillaries being filled with FITC‐BSA, resulting in multiple patchy areas devoid of perfusion. Significant neutrophil (green; PE+anti‐Gr‐1ab) accumulation occurs in IR‐injured hearts. Adherent neutrophils are not the main contributors to compromising blood flow as widespread FITC‐BSA‐perfused capillaries are visible where they are present. Platelet (red; APC+anti‐CD41ab) recruitment is also extensive in injured hearts and they mainly appear as aggregates. These microthrombi occupy and follow the contours of a significant length of the capillary. Co‐localisation (yellow) indicates aggregates are often, but not always, comprised of both neutrophils and platelets. Scale bar = 100 μm (c) Snapshots from a sham heart showing a ‘patrolling’ neutrophil (dotted line). (d) Snapshots from an IR‐injured heart in which the circles outline a circulating neutrophil (green) unable to move downstream of a capillary occluded by a platelet thrombus (red). (e) A large diameter epicardial artery, that was downstream of the LAD artery ligature site, is demarcated by dotted lines. Neutrophils and platelets circulated through it at very high velocities—note the fluorescent streaks they leave behind (arrows in upper panel). Large neutrophil‐rich (green), platelet‐rich (red) and mixed (yellow) aggregates passed through this vessel immediately after the LAD artery was untied, presumably embolising from behind the ligature site. The same circulating aggregate is shown in the left and right panels. Reproduced from Kavanagh et al., 2019a (new Figure 3e)

To end, this review discusses a somewhat less complicated method developed in our own lab to image the IR‐injured beating mouse heart (Kavanagh et al., 2019a). Moreover, it is the first paper to detail a plethora of microcirculatory perturbations taking place in the aftermath of reperfusion injury, again induced by reversible LAD coronary artery ligation. A small 3D‐printed ring, with internal and external diameters of 2.25 and 4 mm, respectively, was lowered onto the left ventricle using a micromanipulator and permanently fixed to the heart using a thin layer of clinical‐grade Vetbond (Figure 3a). Unlike previous methods, we used Nipkow spinning disk confocal microscopy with minimal exposure times in order to capture as many frames as possible during any given amount of time. In conventional laser scanning confocal systems, the excitation laser beam passes through a single pinhole, to become a focussed beam that illuminates a small spot on the specimen. This then scans in a raster pattern across the specimen to form the confocal image. However, because of the time required to acquire an image, laser scanning confocal systems are not suitable for imaging moving tissue or dynamic events. In contrast, spinning disk systems use hundreds of pinholes arranged in spirals on a disk which rotates at high speeds allowing approximately 1000 beams to scans across the area to build the image. This vastly improves the speed of image acquisition allowing for imaging of fast dynamic processes whilst considerably reducing blur, phototoxicity and photobleaching. When applied to the beating heart, both in‐ and out‐of‐focus images were captured within the video. Captured frames that were not in focus due to motion could be disregarded and only in‐focus frames retained using an in‐house software tool, Tify. This was capable of processing large image stacks and performing automated frame removal based on whether or not they contained motion artefacts (Kavanagh et al., 2019b). We did not aim to minimise motion as extensively as others as it was not necessary in order to obtain real‐time videos that were of a good enough quality to both image and subsequently quantitate a variety of different dynamic and static microvascular perturbations. More importantly, we wanted to limit compromising the normal beating and microvascular perfusion of the heart as much as possible.

Using this method, we were the first to not only monitor neutrophil recruitment, but also simultaneously image endogenous platelet activity. Different colour fluorescent dyes, conjugated to specific cell surface non‐function blocking antibodies, were systemically injected. Interestingly, even in healthy sham hearts, adherent neutrophils were noted primarily within the coronary capillaries, but platelet activity was absent (Figure 3b). However, IR injury induced a marked and significant increase in individual neutrophil adhesion, although clusters were also identified. Adherent neutrophils appeared at first glance to be completely stationary. However, detailed analysis of videos demonstrated that some neutrophils, in both healthy and injured hearts, were actually moving short distances or ‘patrolling’ the length of the capillary (Figure 3c), a phenomenon previously described by Jung and colleagues for monocytes in the heart (Jung et al., 2013). The presence of platelet aggregates or microthrombi was most remarkable in the IR‐injured microvasculature. These were not noted in the larger blood vessels but formed primarily within coronary capillaries, often occupying significant lengths of the vessel. Numerous smaller and more rounded platelet aggregates were also found dispersed within the field of view (Figure 3b).

This study was also the first to correlate thromboinflammatory events with perfusion in the myocardium using FITC‐conjugated to albumin (BSA). An extensive network of FITC‐BSA‐perfused capillaries was observed in healthy hearts, paralleling the arrangement of muscle fibres, with cross connections along their length. In contrast, IR injury was associated with multiple areas in which FITC‐BSA did not perfuse. This resulted in patchy areas that appeared devoid of any microvasculature, indicating poor functional capillary density. Indeed, in some fields of view, up to half the imaged area appeared non‐perfused (Figure 3b). Furthermore, the structured parallel arrangement of capillaries was lost with the microvasculature appearing more disorganised. Interestingly, medium‐sized vessels were still readily visible and well perfused in injured hearts. These non‐perfused areas did not correspond with neutrophil adhesion, but rather predominantly matched areas in which the platelet microthrombi were present. These were occlusive and often identified upstream of areas in which no FITC‐BSA was observed. The occlusive nature of platelets was also demonstrated in intravital videos whereby a circulating neutrophil could be observed to stop mid‐flow by an occlusive aggregate of platelets (Figure 3d). The implantation of a stent in patients with MI undergoing a PCI is frequently accompanied by the atheroma and thrombus from the culprit epicardial artery becoming dislodged. This can cause microvascular occlusions, leading to further reductions in myocardial reperfusion. Interestingly our model was able to replicate this phenomenon. Significantly large neutrophil‐rich, platelet‐rich and mixed aggregates were identified circulating through an epicardial artery immediately after the LAD ligation was untied, presumably embolizing from behind the ligature site (Figure 3e).

Our model was also used, for the first time, to image exogenously administered haematopoietic stem/progenitor cell (HSPC) cell trafficking to the heart in vivo at a cellular level and, importantly, ascertain whether vasculoprotection was a critical therapeutic mechanism. We focused on cellular therapy as there has been intense interest in their use for cardiovascular diseases and we had also previously shown HSPCs to reduce inflammatory events in other vascular beds (Kavanagh et al., 2013). Even through cellular therapy has progressed rapidly to clinical trials in patients with heart disease, it is agreed that more basic research is required to understand their mechanisms of action and thus improve on their modest clinical success. A four‐fold increase in systemically injected HSPCs circulating through the IR‐injured heart was observed, but this increased homing did not result in any dramatic local HSPC retention, which remained low. This observation was potentially troublesome, as the efficacy of cellular therapy has been thought to be dependent on sufficient retention within injury sites (Chavakis et al., 2008). However, we showed that despite limited local retention, HSPCs afforded rapid and remarkable vasculoprotection in the injured heart, limiting both neutrophil and platelet adhesion and thus improving functional capillary density and reducing infarct size.

## 6 CONCLUDING REMARKS

The importance of the coronary microcirculation for cardiovascular diseases, particularly after an MI, is increasingly recognised. However, since direct visualisation of the human heart microcirculation clinically is not possible at present, our knowledge of what actually goes wrong in it remains limited. Recent major technological advances in microscopy, the development of elegant stabilisation tools and the ability to label vascular cells using reporter mice or antibodies has meant intravital imaging of the coronary microcirculation in a beating heart in an experimental setting is no longer a far‐fetched idea. Clearly, there are still some limitations in terms of what can be imaged and when. Studies to date are short‐term and performed in terminally anaesthetised animals. Indeed, it is important to consider the impact of anaesthesia on vasoregulation and reducing the heart rate. However, since close contact of the microscope objective lens with the heart, and heart stabilisation, is required for imaging, the use of anaesthesia and surgery is not avoidable. The study by Jung and colleagues did make some progress in longitudinal imaging of the beating heart by developing an in‐house minimally invasive endoscopic imaging probe. However, this probe is not routinely available and the success rate of imaging the same mouse twice was not particularly expanded upon in their paper (Jung et al., 2013). Imaging is also currently restricted to the superficial vasculature—even multiphoton imaging of the beating heart can only acquire z‐stacks up to depths of 100–150 μm, with progressive deterioration of image quality with increasing depth. It remains to be seen whether future advances can increase imaging at even greater depths and improve spatiotemporal resolution of specific trafficking cells within a constantly moving organ.

Nevertheless, it remains important to maximise on currently available advanced technology to characterise in detail the actual perturbations that take place in this microvascular bed in the context of various cardiovascular and also increase our understanding of universal and/or site‐specific mechanisms governing these events. Going forward, animal models that incorporate a co‐morbidity, such as age, diabetes, obesity and hypertension is essential, as these risk factors are commonplace in patients with MI and may increase the susceptibility of the coronary microcirculation to greater damage post‐MI. Moreover, we should exploit this methodology to develop and test strategies that could potentially confer therapeutic benefit at the level of the coronary microcirculation and accelerate the much‐needed clinical progress in this area.

## ACKNOWLEDGEMENT

We are grateful to the British Heart Foundation (PG/14/92/31234) for supporting the research used to generate some of the images in Figure 3.

## Data Availability

Data sharing is not applicable to this review article as no new data were created or analysed in this study.
